# *Aniba canelilla* (Kunth) Mez (Lauraceae): A Review of Ethnobotany, Phytochemical, Antioxidant, Anti-Inflammatory, Cardiovascular, and Neurological Properties

**DOI:** 10.3389/fphar.2020.00699

**Published:** 2020-05-26

**Authors:** Fabio J. C. Souza-Junior, Daniele Luz-Moraes, Felype S. Pereira, Mayra A. Barros, Luanna M. P. Fernandes, Letícia Y. Queiroz, Cristiane F. Maia, José Guilherme S. Maia, Enéas A. Fontes-Junior

**Affiliations:** ^1^Laboratório de Farmacologia da Inflamação e do Comportamento, Universidade Federal do Pará, Belém-PA, Brazil; ^2^Programa de Pós-Graduação em Ciências Farmacêuticas, Instituto de Ciências da Saúde, Universidade Federal do Pará, Belém-PA, Brazil

**Keywords:** *Aniba canelilla* (Kunth) Mez, folk medicine, biological activities, toxicity, 1-nitro-2-phenylethane, methyleugenol

## Abstract

*Aniba canelilla* (Kunth) Mez, popularly known as “casca preciosa” (precious bark), falsa canela (cinnamon-scented) Casca-do-maranhão (bark of maranhão), and Folha-preciosa (precious leaf), is an aromatic species of the Lauraceae family, widely distributed in the Amazon region. In traditional medicine, it is indicated for the treatment of a great diversity of diseases, including digestive, respiratory, inflam]matory, painful, and central nervous system disorders, it is administered mainly in the form of tea or decoction orally. Its essential oil is referred to as a natural antioxidant for food preservation and disease control, showing great potential for use in the cosmetics, perfumery, and pharmaceutical products sector. The present review aimed to discuss critically and comprehensively the ethnobotanical characteristics, phytochemical constitution, and scientifically tested biological properties of *A. canelilla*, systematizing the knowledge about the species and proposing new perspectives for research and development. The chemical composition of *A. canelilla* includes 1-nitro-2-phenylethane, metyleugenol, eugenol, safrol, anabasin, anbin, tannin, α-pinene, b-pinene, b-felandren, b-caryophyllene, b-sesquifelandren, p-cymene, linalool, α-copaene, and spatulenol. Researches with ethanolic extracts, essential oils, and major constituents (1-nitro-2-phenylethane and metyleugenol) have revealed antioxidant, antinociceptive, anti-inflammatory, cardio-modulating, hypotensive (vasorelaxant), hypnotic, anxiolytic, anticholinesterase, and antibiotic properties (trypanomicidal, leishmanicidal, and antifungal). Some of these effects are potentially beneficial for aging-related diseases treatment, such as cardio and cerebrovascular, chronic inflammatory, neurological, and degenerative diseases. However, it is necessary to advance in the research of its clinical use and development of therapeutic products.

## Introduction

The increase in human longevity, as well as population growth, is a result of its social and technological evolution. This phenomenon, however, represents a growing public health challenge due to the proportional increase in the incidence of chronic inflammatory, neurological, and cardiovascular diseases. Such pathological conditions often require chronic polymedication which are sometimes marked by intense adverse reactions and are not always effective, leading to deterioration of patients' quality of life or discontinuation of therapy. There are still cases where current therapy consists only of palliative measures, with little effect on disease progression (Boccardi, 2019). This reality drives the scientific community in search of safer and more effective drugs.

Finding a solution for the future, however, may be related to our ability to investigate the past, valuing all traditional knowledge about plant species with therapeutic potential but lacking studies on their safety and effectiveness. In fact, medical plants have been a valuable source of herbal medicines and bioactive molecules for drug development (Samy et al., 2008). Brazilian forests, especially the Amazon rainforest, represent the largest source of plant species in the world, many of which have medicinal benefits. Along with such biodiversity, the Brazilian population also has a very rich folk medicine, preserved by traditional communities, which provides valuable information, useful to the selection of species for pharmacological screening (Hegde et al., 2014; Moraes et al., 2019).

Among the plants inserted in the Amazonian culture, *Aniba canelilla* (Kunth) Mez [syn. *Aniba elliptica* A.C. Sm., *Cryptocarya canelilla* Kunth, and others] stands out for its diversity economic and medicinal applications. This species of Lauraceae is an aromatic medium-sized tree, native from solid ground and semi-deciduous forests of South America, known as *casca-preciosa* (precious bark), *falsa-canela* (fake cinnamon), *casca-do-maranhão* (maranhão bark), and *folha-preciosa* (precious leaf), (Gottlieb and Magalhães, 1960b; Kubitzki and Renner, 1982; Ribeiro et al., 1999; Franciscon et al., 2018; Tropicos.org, 2019). Among its characteristics, the cinnamon-like aroma stands out, is even linked to the history of its discovery. Around Brazil discovery period, when Portuguese and Spanish navigators entered the Amazon River in search of cinnamon (*Cinnamomum verum* J.Presl) thought they had found a lot of this plant, instead what they had found was Casca-preciosa trees (Pinto, 1995). In folk medicine, this species is often used to treat painful and inflammatory conditions, gastrointestinal disorders, and neurological and psychiatric diseases, in addition to the treatment of infections (Maia et al., 2001; De Filipps et al., 2004; Sousa et al., 2009; Manhães et al., 2012). Some of these applications have been studied, elucidating their main constituents and ratifying their anti-inflammatory, antinociceptive, antihypertensive, and antioxidant potential, among others.

Nevertheless, it is noted that much remains to be elucidated about the therapeutic potential of *A. canelilla*, including the safety of its use, medical applications, and mechanisms of action. In this sense, the present study aims to review critically and comprehensively the current knowledge about the ethnobotany, phytochemical, pharmacological, and toxicological characteristics of *A. canelilla* and its derivatives (extracts, essential oil, and major constituents), identifying perspectives for new research and exploring its potential for neurological and cardiovascular diseases treatment (Graphical abstract—**Figure S1**). Data collection on *A. canelilla* was carried out electronically, based on articles published in peer-reviewed journals, abstracts published in conference proceedings, theses, and ethnobotanical textbooks. The research was carried out in the Google Scholar, Science Direct, Scopos, and PubMed databases.

## Ethnobotany

### Taxonomy and Botanical Aspects

Lauraceae is a family of trees and shrubs recorded since the Cretaceous period, which includes 52 genera and approximately 2,500 to 3,500 species, distributed in tropical and subtropical areas, as well as in temperate climates, as America, tropical Asia, Australia, and Madagascar (Van der Werff, 1991; Kubitzki et al., 1993; Hu et al., 2007). This family gathers medium to large trees, but some species have characteristics of shrubs or small trees, with a diameter between 1 and 5 cm and their height rarely exceeds 8 m (Ribeiro et al., 1999). Several species of Lauraceae have economic use, providing wood, essential oils, and chemical compounds, relevant to civil construction and for the food and pharmaceutical industries (Barbosa et al., 2012). In Brazil, 24 genera and 441 species have been identified (Quinet et al., 2015). Some genera of Lauraceae are highlighted by having aromatic species, that is, plants that produce essential oils (EO) or resins rich in volatile compounds, as *Aniba*, *Nectandra*, *Ocotea*, *Licaria*, and *Dicypellium* (Marques, 2001).

As previously mentioned, the species *A. canelilla* is an aromatic plant with a characteristic odor, which is easily confused with cinnamon. Historically this characteristic is portrayed in the expeditions of Pizzarro and Orellana, in 1540, to the Amazon River, and Humbolt and Bonpland to the Orinoco River in 1800. In both cases, it was considered that excellent sources of the coveted spice were found, when in fact they found *Casca-preciosa* (Naranjo et al., 1981; Pinto, 1995; Lahlou et al., 2005). Its proper botanical designation was made by Carl Kunth, a German botanist, from samples collected by Humbolt and Bonpland. In his description, the botanist emphasized the aromatic characteristic of the plant and its similarity to cinnamon. Initially called *Aniba canelilla* by Kunth, it was later registered and published by Carl Mez (1889), being officially identified as *Aniba canelilla* (Kunth) Mez (Gottlieb and Magalhães, 1960b; Almeida et al., 2017).

This prominent tree reaches up to 35 m in height and has a retinal trunk, with a diameter between 40 and 60 cm. Its branches are glabrous and lenticelated, with 8 to 16 cm leaves (thin petioles, grayish and dense garment, and secondary veins flattened and slightly prominent at the bottom), small flowers, and oblong capsular fruit (De Siqueira et al., 2010).

### Distribution and Traditional Uses

**Figure 1:**
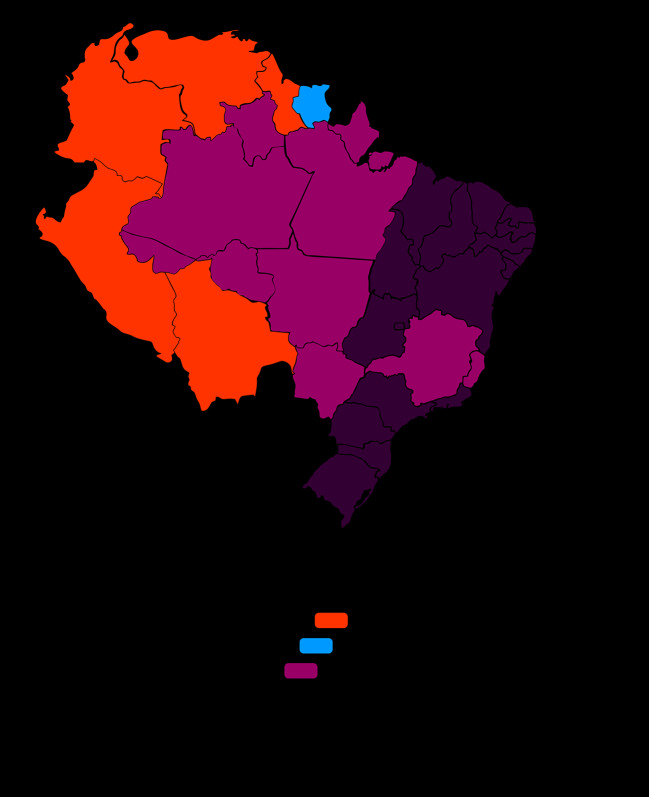
Distribution of *Aniba canelilla* in South America. Blue color in cartogram indicates coutries where the *A*. *canelilla* tree is native, Orange color indicates countries where its native presence is doubtful, and the dark green color highlights the states of Brazil where it is native. Adapted from Quinet et al., 2015.

Native to the plains and solid ground forests of the Amazon rainforest and Guyana, *A. canelilla* is adaptable to different types of soils, including regions of solid or semi-deciduous forests, lowland areas, rocky outcrops and rocky soils, poorly drained soils, and clay and sandy soils (Franciscon et al., 2018). This species extends from the Antilles, Guyana high lands to the dry lands of Brazil. In fact, it presents a wide distribution in the Amazon region, which predominates from the East of French Guiana, Suriname, Venezuela, Colombia to the Peruvian Amazon, rarely found in Central America (Kubitzki and Renner, 1982; Van der Werff, 1991; Ribeiro et al., 1999; Quinet et al., 2015; **Figure 1**). **Table 1** summarizes the various popular names attributed to *A. canelilla*, by geographic location.

**Table 1:** Popular names of *Aniba canelilla* according to its geographical location.

Popular name	Geographic location	Reference
Amapaima	Guiana	De Filipps et al., 2004
Ashmud	Guiana	De Filipps et al., 2004
Canela	Brazil: Pará	Herrero-Jaurégui et al., 2009
Canelão	Brazil: Manaus	Costa, 2013
Canelón	Bolivia^1^, Peru^2^	Fournet et al., 1994^1^; Organismo Andino de Salud, 2014^2^
Casca-do-maranhão	Brazil: Maranhão	Madaleno, 2011
Casca-preciosa	Brazil: Pará^1^, Manaus^2^, Rondônia^3^ Guiana^4^	Elisabetsky and Castilhos, 1990^1^; Bitencourt et al., 2014^1^; Santos et al., 2016^1^; Lima et al., 2004^2^; Silva et al., 2011^3^; De Filipps et al., 2004^4^
Chorecho	Bolivia	Fournet et al., 1994; Guze et al., 2014
Guarimán	Venezuela	Francisco and Ortega, 1994
Koto chojlla	Bolivia	Thomas and Vandebroek, 2006
Pokaneragi	Bolivia	Thomas and Vandebroek, 2006
Preciosa	Brazil: Amazonas^1^, Maranhão^2^, Pará^3^	Quignard et al., 2003^1^; Madaleno, 2011^2^; Branch and Silva, 1983^3^; Almeida et al., 2013^3^; Lima et al., 2011^3^
Showoyaja	Bolivia	Thomas and Vandebroek, 2006

Superscript 1-4, numbering is used to correlate the items in the same row as columns 2 and 3, pointing out the reference (column 3) corresponding to the information (column 2).

*A. canelilla* is present in several aspects of the culture of these peoples. Its wood is used for civil construction (Lupe, 2007) and its EO and extracts are consumed as spices, cosmetics, and perfumes, as the product known as “Cheiro-de-santarém” (Maia et al., 2011; Araújo et al., 2007; Lorenzi and Matos, 2008; Barbosa et al., 2017).

In folk medicine, multiple ethnopharmacological indications have been found, with variations in the form of use. The most frequently mentioned form was the infusion of the barks, used to treat stomach inflammation, pain, and ulcers (De Almeida et al., 2013). Interestingly, the plant has been cited as a natural soothing, and still cited by indigenous communities as tranquilizer (Oger et al., 1994; Almeida et al., 2013; Santos et al., 2016), in contrast to some studies that point a stimulating and antidepressant effect (Rodrigues and Carlini, 2006; Bitencourt et al., 2014). There were also indications for the treatment of colds, cough, headache, and nausea (Taylor, 2005). Topical applications of bark extracts have been cited for the treatment of skin diseases, injuries, and infections (Mors et al., 2000; Taylor, 2005). *A. canelilla* EO (AcEO) also has a wide range of indications. According to Barbosa et al. (2017), the AcEO has therapeutic properties similar to those attributed to the infusions of bark, leaves, and branches, and there are also citations of its use to treat acne, dermatitis, fever, and various types of infections and injuries (Taylor, 2005). Another interesting finding is the use of smoke produced by barks burning, by the Tiryó Indians (Suriname), to treat diarrhea (De Filipps et al., 2004). References of the use of this species as analgesic, anti-inflammatory, antispasmodic, anti-anemic, anti-dysenteric, and antibiotic are also frequent. Additional uses of *A. canelilla* in folk medicine are summarized in **Table 2**.

**Table 2:** Traditional uses of *Aniba canelilla*.

Popular indication	Part of the plant	Preparation	Administration	Reference
Arthritis, dyspepsia, infection, weakness, chest stimulant	Bark Leaves	Not stated	Not stated	Botsaris, 2007
Malaria	Bark^1,2^ Leaves^1,2^ Seeds^1^	Not stated	Not stated	Quignard et al., 2003^1^; Botsaris, 2007^2^
Sinusitis	Bark	Tea	Oral	Silva et al., 2011
Intestinal colic	Bark^1,2^ Leaves^2^	Not stated	Not stated	Deharo et al., 2001^1^; Botsaris, 2007^2^
Fever	Bark^1,2,3^ Leaves^3^	Maté^1^ Decoction ^2^	Oral	Fournet et al., 1994^1^; De Filipps et al., 2004^2^; Botsaris, 2007^3^
Calmative	Bark	Tea	Oral	Almeida et al., 2013; Santos et al., 2016
Migraine	Bark	Maté	Oral	Fournet et al., 1994
Dysentery	Bark^1,2,3^ Leaves^1^ Seeds^1^	Maté Decocção^2^ Tea^3^	Oral	Quignard et al., 2003^1^; De Filipps et al., 2004^2^; Bitencourt et al., 2014^3^
Diarrhea	Bark	Maté^1^	Oral	Fournet et al., 1994^1^; Thomas and Vandebroek, 2006; Luziatelli et al., 2010
Vomiting	Bark	Not stated	Not stated	Thomas and Vandebroek, 2006
Stomachache	Bark	Decoction^1,3^ Tea^2^	Oral	Elisabetsky and Castilhos, 1990; Bourdy et al., 2000^1^; Deharo et al., 2001; Almeida et al., 2013^2^; Thomas and Vandebroek, 2006; Costa, 2013^3^
Postpartum recovery	Bark	Not stated	Not stated	Deharo et al., 2001
Syphilis	Bark Seeds Leaves	Not stated	Not stated	Quignard et al., 2003
Inflammation	Bark^1,2^ Leaves^2^	Tea	Oral	Almeida et al., 2013^1^; Organismo Andino de Salud, 2014^2^
Pain	Bark Leaves	Not stated	Not stated	Organismo Andino de Salud, 2014
Gout, catarrh	Bark	Tea	Oral	Bitencourt et al., 2014
Ulcer	Bark	Tea	Oral	Almeida et al., 2013
Against human hookworm	Bark	Extract (cooblation)	Not stated	Marques, 2001
Alzheimer's disease	Not stated	Not stated	Not stated	Madaleno, 2011
Antidepressant/stimulant	Bark ^1^	Tea^1^	Oral	Rodrigues and Carlini, 2006; Bitencourt et al., 2014^1^

Superscript 1-4, numbering is used to correlate the items in the same row as columns 2, 3 and 5, pointing out the reference (column 5) corresponding to the information (column 2).

## Phytochemical Composition

### Extracts

There are few studies dedicated to elucidate the phytochemical composition of *A. canelilla* extracts and carried out studies present only screens of the metabolite classes. For example, the study conducted by Mesquita et al. (2014) demonstrated the presence of phenolic compounds, tannins, flavonoids, and saponins in ethanol extracts obtained from leaves and branches. Despite the typical presence of benzyltetrahydroisoquinoline alkaloids in Lauraceae species, such as reticulin, coclaurine, and noranicanine (Oger et al., 1992; Oger et al., 1993; Custódio and Veiga Junior, 2014), the evaluation of the ethanolic extracts of *A. canelilla* did not detect alkaloids, as well as steroids and triterpenes.

### Essential Oil

AcEO, unlike other extracts, has been well characterized. It is a yellowish-brown oil, denser than water, which have the characteristic aroma of the specie. It can be extracted from the trunk wood, branches, leaves, thin stems, and barks, by steam distillation or hydrodistillation, using a Clevenger-type apparatus, with a yield between 0.2 to 1.3% (Gottlieb and Magalhães, 1960a; Oger et al., 1994; Taveira et al., 2003; Da Silva et al., 2007; Silva et al., 2009; Silva et al., 2014; Barbosa et al., 2017; Giongo et al., 2017). Several factors, such as humidity and incidence of light, physiological, and geographical variations, environmental conditions, and genetic factors, can influence the production of AcOE. The available studies demonstrate higher oil yield in rainy periods, in the initial periods of development of plant organs, and in conditions where there is a greater supply of light. Regarding the parts of the plant, its yield of the stem is higher in the dry seasons. Leaf yield, however, does not appear to be influenced by such factors (Taveira et al., 2003; Manhães et al., 2012).

**Figure 2:**
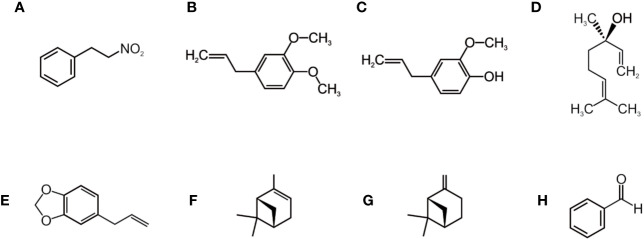
Substances present in the *Aniba canelilla* essential oil: 1-nitro-2-phenylethane **(A)**, methyleugenol **(B)**, eugenol **(C)**, linalool **(D)**, safrole **(E)**, α-pinene **(F)**, β-pinene **(G)**, benzaldehyde **(H)**. Adapted from: Sugimoto et al. (2017), Sipe et al. (2014), Tsuchiya (2017), Rianjanu et al. (2018), Silva et al. (2012).

Its chemical composition has two main constituents, 1-nitro-2-phenylethane (1N2PE), the first known natural nitro derivative which is generally pointed as the major component and is responsible for the cinnamon-like odor characteristic of the species, and methyleneugenol (Gottlieb and Magalhães, 1959; Gottlieb and Magalhães, 1960a; Oger et al., 1994; Maia et al., 2001; Taveira et al., 2003; Lima et al., 2004; Da Silva et al., 2007; Maia and Andrade, 2009; Silva et al., 2009; Sousa, 2011; Manhães et al., 2012; Vale et al., 2013; Almeida et al., 2017; Farias et al., 2017; Giongo et al., 2017). These substances were identified both in the stem wood, bark, and leaves. Other identified constituents are eugenol, safrole, anabasine, anbin, tannin, α-pinene, b-pinene, b-felandren, b-caryophyllene, b-sesquifelandren, p-cymene, linalool, α-copaene, and spatulenol (Sangwan et al., 2001; Figueiredo et al., 2008). The main chemical constituents present in AcEO are listed in **Figure 2**.

As well as the oil yield, its composition also suffers seasonal influence. In the rainy period, the 1N2PE concentrations reach near 95%, while methyleugenol remain below 18%. In the dry period, 1N2PE decrease to less than 40%, while methyleugenol reaches 45%. The greatest value of 1N2PE (95.3%) and the least value of methyleugenol (0.2%) were observed in the leaves, during the rainy season (Taveira et al., 2003). Silva et al. (2009), otherwise, found no variation in 1N2PE concentration from the rainy to the dry seasons in samples collected on Reserva Adolpho Ducke, AM, Brazil. Additionally, methyleugenol was not detected in oils (Silva et al., 2009). Regarding the parts of the plant, Taveira et al. (2003) has found higher percentage of 1N2PE in leaves, while Manhães et al. (2012) found higher concentrations in stems. The content of 1N2PE and methyleugenol in leaves and fine stems are comparable to the content observed in the trunk wood and bark of *A. canelilla*. Thus, the extraction of essential oil from leaves and branches becomes an alternative to prevent the overthrow of the trunk to produce essential oils from this specie (Barbosa et al., 2017).

### 1-Nitro-2-Phenylethane

1N2PE (C_8_H_9_NO_2_, MW 151.16) is a nitro compound formed through the biotransformation of phenylalanine by enzymes of the CYP superfamily (cytochrome P450) (Gottlieb and Magalhães, 1959; Gottlieb, 1972; Matsuo et al., 1972; Wittstock and Halkier, 2000). It is also found in two other species, *Ocotea pretiosa* (Nees & Mart.) Mez (Lauraceae) (Gottlieb and Magalhães, 1959), present in São Paulo, Rio de Janeiro, Minas Gerais and Santa Catarina (Brazil) states, where it is known as *Canela-sassafrás*, and *Dennettia tripetala* G. Baker (Annonaceae) (Okogun and Ekong, 1969; Oyemitan et al., 2013). Due to the characteristic odor generated by the nitro compound, *O. pretiosa* can be confused with *A. canelilla*. EO samples from *O. pretiosa* collected in the warmer regions Brazilian southeastern showed 1N2PE and methyleugenol with main constituents (Gottlieb and Magalhães, 1959), while samples collected in the south, colder region, showed higher proportions of safrole (Gottlieb and Magalhães, 1960a).

### Methyleugenol

Chemically referred to as 1-allyl-3,4-dimethoxybenzene (C_11_H_14_O_2_, MW 178.23), methyleugenol is a yellowish oily compound with the characteristic of clove aroma and bitter taste, present in a wide variety of EOs. It is soluble in several organic solvents and insoluble in water. Methyleugenol is formed from the methylation of eugenol, another minor phenolic compound found in the AcEO (Gottlieb and Magalhães, 1959; Lahlou et al., 2004; Burdock, 2005; Barbosa et al., 2017). Concerning its occurrence in the parts of the plant, it is mainly concentrated in the trunk wood, where it has already been quantified at the concentration of 45.8% (Taveira et al., 2003). In the state of Pará, Brazil, it is considered a chemical marker for the AcEO, is also a significant constituent in other Lauraceae species, such as *O. pretiosa* from Southeastern Brazilian States (Gottlieb and Magalhães, 1960a; Silva et al., 2009; Manhães et al., 2012).

## Biological Properties

Traditional communities attribute a variety of therapeutic properties to *A. canelilla*, being applied to the treatment of painful and inflammatory conditions, gastrointestinal and respiratory diseases, microbial infections, and parasitosis, in addition to neuropsychiatric disorders, as detailed above. Therefore, based on the medicinal culture of these people, much has been explored in scientific research, even though most of these studies have little depth. The studies identified in this review addressed mainly the toxicity antioxidant, antinociceptive, anti-inflammatory, cardiovascular, and neuropharmacological activities of *A. canelilla* EO and extracts in murine or *in vitro* models. Evidence of anticholinesterase and antibiotic activity was also verified (**Table 3**).

**Table 3:** Biological activities of *Aniba Canelilla* extracts, essential oil, and 1-nitro-2-phenylethane.

Experimental model	Part of the plant	Evaluated drug	Dose/Conc. interval	Activity	References
Nociception in mice * Writhing test* * Formalin test* * Hotplate test*	Bark	1N2PE	15–50 mg/kg	Antinociceptive	Lima et al., 2009
Inflammation in rats * Dextran paw edema* * Carrageenan paw edema* * Croton oil ear edema*	Bark	1N2PE	25–50 mg/kg	Antiedematogenic	Vale et al., 2013
*In vitro* isolated rat aorta	Bark	EO	1–600 µg/mL	Vasorelaxant	Lahlou et al., 2005
	Bark	1N2PE	1–300 µg/mL		De Siqueira et al., 2010
Normotensive rats	Bark	EO	1–20 mg/kg	Bradycardic and hypotensive	Lahlou et al., 2005
	Bark	1N2PE	1–10 mg/kg		De Siqueira et al., 2010
Hypertensive rats	Bark	EO 1N2PE	10–20 mg/kg 5–10 mg/kg	Bradycardic and hypotensive	Interaminense et al., 2010; Interaminense et al., 2013
Neuropharmacological evaluations in mice * Sleeping time* * PTZ-induced convulsion* * Elevated plus-maze*	–	1N2FE	50–400 mg/kg 25–200 mg/kg 5–20 mg/kg	Hypnotic, Anticonvulsant Anxiolytic	Oyemitan et al., 2013
*In vitro* acetylcholinesterase activity assay	Trunk wood	EO 1N2PE	0.01–1,000 ng/spot	Anticholinesterase	Silva et al., 2014
Tripomastigotes culture * Trypanosoma evansi*	Wood	EO 1N2PE	0.5–2.0%	Trypanocide	Giongo et al., 2017
*In vitro* antileishmanial assay * Leishmania amazonensis*	Leaves	EO	40 µg/mL^*^	Leishmanicide	Silva et al., 2009
*In vitro* microdilution test * Candida albicans* * C. tropicalis* * C. parapsilosis* * Aspergillus. fumigatus*	Bark	1N2PE	170 µg/mL^**^ 360 µg/mL^**^ 720 µg/mL^**^ 1500 µg/mL^**^	Antifungal	Oger et al., 1994
Challerger test * Candida albicans* * Didymella bryoniae*	Leaves Branches	EO	2 mg/mL	Antifungal	Silva, 2012

*Median inhibitory concentration (IC_50_).

**Minimum inhibitory concentration (MIC).

EO, essential oil; 1N2PE, 1-nitro-2-phenilethane; ETEX, ethanolic extract; PTZ, pentylenetetrazol.

### Toxicity

Characterizing the safety of using a therapeutic agent is as important as ensuring its effectiveness, being a requirement for the registration of herbal agents. The present review identified *in vitro*, and *in vivo* toxicological tests that aimed to characterize acute and subacute toxicity of *A. canelilla* derived products.

The cytotoxicity of AcEO and its two main constituents was tested by Giongo et al. (2017) in primary lymphocyte culture. AcEO, 1N2PE, and methyleugenol were tested in concentrations of 0.5%, 1%, and 2%, producing no cytotoxicity on those cells. However, the samples treated with a mixture of 1N2PE and methyleugenol in the concentration of 2% showed an average reduction of 21% in cell viability, pointing to the potential risk of the association of these constituents in high concentrations. In the brine shrimp bioassay, Silva et al. (2007) performed the analysis of plant samples collected in the Brazilian states of Para and Amazonas in rainy season. Methanolic extracts and EO from the trunks wood were tested, as well as 1N2PE isolated from EO. Products presented medium lethal concentration (LC_50_) of 91.38 ± 7.20 μg/mL, 21.61 ± 1.21 μg/mL, and 20.37 ± 0.99 μg/mL, respectively, highlighting that 1N2PE showed equivalent activity to EO, and methanolic extract presented toxicity about four times lower.

AcEO oral toxicity was tested in an acute and subacute pattern of administration in murine models by Sousa et al. (2009). In the acute evaluation, performed with mice, the authors presented a medium lethal dose (LD_50_) of 720 ± 66.4 mg/kg, despite declaring that the “study does not show any toxic symptoms, changes in behavior or mortality at the tested doses.” For 1N2PE, LD50 of 712 ± 17.39 mg/kg for oral administration (Lima, 2008) and 490 mg/kg for intraperitoneal (ip) administration were identified in mice (Oyemitan et al., 2013).

Subacute toxicity (30 days of treatment) was tested in rats, with doses equivalent to 5% (36 mg/kg) and 10% (72 mg/kg) of LD_50_, evaluating the incidence of deaths and behavioral, hematological (red cells, hematocrit, hemoglobin, MCV, HCM, MCCH, leukocytes, segmented cells, lymphocytes, and monocytes), biochemical (glucose, urea, creatinine, triglycerides, cholesterol, ALT, AST, and alkaline phosphatases), and histopathological markers. AcEO did not promote deaths in tested doses, nor did it promote significant changes in animals' behavior, as well as in the biochemical and hematological parameters tested. The vital organs showed no histological changes, except for the liver, which presented necrotic foci, apoptotic cells, and mononuclear infiltrate. There was also evidence of tissue regeneration, which, together with the absence of changes in biochemical markers, indicates adaptation of the organ to aggression. Together, these findings classify AcEO and 1N2PE as class 4 xenobiotics (low toxicity) (OECD, 2001).

### Antioxidant Activity

The genesis and evolution of chronic degenerative diseases, as well as several pathologies, are strongly related to the imbalance between reactive oxygen species (ROS) production and the body's enzymatic and non-enzymatic antioxidant agents, a state called oxidative stress. Several EOs are recognized for their antioxidant potential, used for food preservation, but it also has pharmaceutical interest (Ruberto and Baratta, 2000; Miliauskas et al., 2004; Mimica-Duric et al., 2004). Such properties have been associated with the presence of phenolic compounds, such as flavonoids, phenolic acids, and phenolic diterpenes in aromatic plants. Studies carried out with *A. canelilla* highlight its potential, related to the presence of antioxidant phenolic compounds and coumarins, for the development of cosmetic, anti-corrosion products for the metallurgical industry and pharmaceutical products (Da Silva et al., 2007; Silva, 2012; Mesquita et al., 2014; Farias et al., 2017; Barros et al., 2018).

Despite the potential of its phytochemical constituents, until now only chemical tests have been used to evaluate the antioxidant capacity of *A. canelilla* derivatives, based on its ability to eliminate DPPH (2,2-diphenyl-1-picrylhydrazyl) radicals, an assay aimed to screen non-enzymatic antioxidant agents candidate scavenger activity, using the Trolox (6-hydroxy-2,5,7,8-tetramethylchroman-2-carboxylic acid), ascorbic acid, and/or quercetin as activity patterns. Da Silva et al. (2007), using Trolox as standard, demonstrated an DPPH inhibition of 32 to 93% for samples of AcEO (110 to 1,400 µg/mL) obtained from Amazonas and Pará state (Brazil) plants. Methanolic extracts (2 to 10 µg/mL) obtained from same plants showed an DPPH inhibition of 25 to 93%, and 11.47 to 63.19% of inhibition was found for 1N2PE (200 to 1000 µg/mL). Such findings suggest a greater antioxidant capacity for the methanolic extract, up to fifty times higher than that of AcEO and equivalent to Trolox. For ethanolic extract of the plant branches and leaves, Mesquita et al. (2014) demonstrated a greater scavenger activity in leaf extract, that showed 34.97% of ascorbic acid activity. This finding was similar to the one found by Silva (2012), who noticed that the ethanolic extract of the leaves had about one-third of ascorbic acid activity. In the same study, the hydroalcoholic extract presented similar results. The results by Martins et al. (2016), on the other hand, demonstrated equivalence between ascorbic acid and the ethanolic extract from the bark of *A. canelilla*. They also demonstrated that the extracts had photoprotective effect.

In this context, considering the preliminary character of these findings and the identification of secondary metabolites, phenolic compounds, and coumarins, related to antioxidant activity and the reduction of the risk of various diseases, in products obtained from *A. canelilla* (Bubols et al., 2013; Mesquita et al., 2014; Serino and Salazar, 2019), we believe it is essential to explore biological models to evaluate its effects on enzymes with oxidizing [nicotinic adenine dinucleotide phosphate oxidase (NOX), nitric oxide synthase (NOS), monoxides, and xanthine oxidoreductase (XO), etc.] and antioxidant [catalase, glutathione (GPx), superoxide dismutase (SOD), etc.] activities, as well as on the formation of ROS and oxidative injury markers (lipid peroxidation, carbonylated proteins, etc.) (Awad et al., 2018) to determine its real benefit for the prevention or treatment of chronic and/or degenerative metabolic, cardiovascular, neural, and other diseases, whose mechanisms of genesis and evolution comprise of an important oxidative component (for review: Uttara et al., 2009; Liguori et al., 2018).

### Antinociceptive and Anti-Inflammatory Activity

Inflammation is another key pathophysiological process in the origin and development of numerous diseases. Considering not only the chronic inflammatory conditions that are increasing in function of aging, but it has also been widely discussed in cardiovascular and neuropsychiatric diseases context, showing clinical benefits with anti-inflammatory drugs use. Pain is involved in worsening the patient's quality of life, requiring non-pharmacological and pharmacological care aimed at its adequate control. In this context, *A. canelilla* has shown promise, since published studies have ratified, in murine models, its antinociceptive and anti-inflammatory properties referred in traditional use.

The study initially conducted by Lima (2008), later expanded and published in two other articles (Lima et al., 2009; Vale et al., 2013), demonstrated antinociceptive and anti-inflammatory activity using a fraction of the AcEO containing 1N2PE with 97.5 to 99% purity. This product was therefore applied to classic models of antinociceptive activity (abdominal contortions, hot plaque, and formalin) and antiedematogency (croton oil ear edema, paw edema by carrageenan or dextran), in addition to performing a theoretical study of functional density calculations. Regarding nociception, 1N2PE showed antinociceptive activity in a dose-dependent pattern (15, 25, and 50 mg/kg) when administered ip. The screening proceeded also revealed that it modulates only peripheral components of nociception, despite apparently involving opioid receptors in its mechanism of action (Lima et al., 2009). In inflammatory context, the results indicate the reduction of edema induced by croton oil, carrageenan, and dextran, with oral doses of 25 and 50 mg/kg (Vale et al., 2013). A possible mechanism related to these effects was pointed out by a conformational study conducted by Vale et al. (2013) who demonstrated that 1N2PE molecule had a favorable configuration for interaction with prostaglandin endoperoxide synthase.

Methyleugenol, the second major compound in AcEO, it also shows evidence of antinociceptive, anesthetic, and anti-inflammatory activity. According to Yano et al. (2006) research, its oral administration (10 mg/kg) reduces the second stage nociceptive response in formalin test. This effect was inhibited by bicuculline, a GABAa receptor antagonist, indicating that its activity would be related to the activation of these receptors. On the other hand, it did not promote any activity on cyclooxygenases 1 and 2. These findings seem to corroborate with Carlini et al. (1981) study, that attributed anesthetic activity to methyleugenol (200 and 300 mg/kg). Additionally, Choi et al. (2010) demonstrated a reduction in the expression of pro-inflammatory (IL-1B, IL-6, TNFα, and iNOS) and increased expression of anti-inflammatory (IL-10, TGF-B) mediators in rat and cell culture models of cerebral ischemia. In view of these results, there is still much to be done in elucidating mechanisms of its antinociceptive and anti-inflammatory properties. Also taking advantage of central nervous system activity evidences to better characterize its potential anti-neuroinflammatory effect.

### Cardiovascular Activities

**Figure 3:**
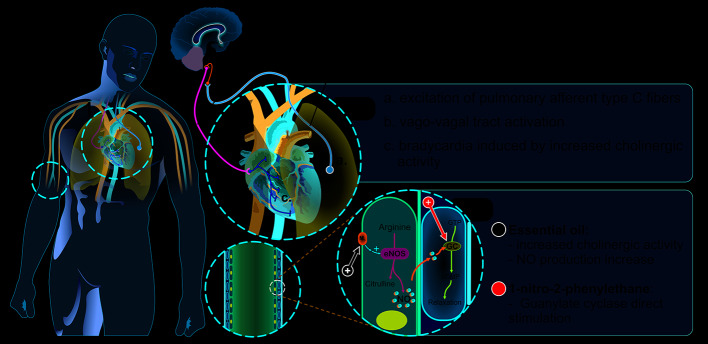
Proposed mechanism for the antihypertensive and bradycardic effects of *Aniba canelilla* essential oil and 1-nitro-2-phenylethane.

Cardiovascular effects of AcEO and its major components were elucidated through a partnership between researchers from the federal universities of Ceará and Pará (Brazil). The work developed by those groups initially demonstrated hypotension and bradycardia being promoted by intravenous (i.v.) injection of AcEO in normotensive rats, triggering in a dose-dependent pattern. According to the authors, such effects were linked, at least in part, to endothelial integrity factors, also identifying the participation of endothelial muscarinic receptors and stimulation of nitric oxide (NO) production, regulated by calcium flow (Lahlou et al., 2005). The same pattern was observed with i.v. administration of a concentrated fraction (98%) of 1N2PE (1, 3, 5, and 10 mg/kg) in normotensive anesthetized rats, indicating that 1N2PE was responsible for the vasorelaxant and bradycardic properties of AcEO (De Siqueira et al., 2010). According to the authors, this effect was a vago-vagal bradycardiac and depressor reflex generated by excitation of pulmonary afferent type C fibers. Also, 1N2PE has a direct effect on the vascular smooth muscle, promoting its relaxation. Thus, the authors demonstrated that the i.v. treatment with AcEO actively promoted vascular relaxation and consequent hypotension, not associated with the sympathetic tone (Lahlou et al., 2005; De Siqueira et al., 2010). In the next research stage, cardiovascular effects of AcEO and 1N2PE were tested in spontaneously hypertensive rats. Under these conditions, the i.v. administration of both AcEO (1, 5, 10, and 20 mg/kg) and 1N2PE (1, 3, 5, and 10 mg/kg) retained the same pattern of biphasic effect, composed of the vago-vagal bradycardic reflex (phase 1) and the direct vasodilatory action (phase 2; **Figure 3**) (Interaminense et al., 2010; Interaminense et al., 2013).

The biochemical pathways involved on those effects were investigated by Brito et al. (2013) who reported that 1N2PE would raise the levels of cyclic guanosine monophosphate (cGMP) in smooth muscle cells, suggesting a possible stimulation of guanylate cyclase (sGC). In addition, blocking the production of cyclic adenosine monophosphate (cAMP) or NO did not interfere with 1N2PE effects. Such evidence reveals the potential of 1N2PE for the treatment of cardiovascular diseases, such as systemic arterial hypertension and pulmonary arterial hypertension, and cerebrovascular diseases, such as stroke.

### Neuropharmacological Activities

Despite the references of *A. canelilla* tea traditional use for the treatment of central nervous system-related conditions, such as Alzheimer's disease and depression, in addition to indication as calming (Rodrigues and Carlini, 2006; Madaleno, 2011; Almeida et al., 2013; Bitencourt et al., 2014; Santos et al., 2016), no studies were identified that explored such properties in this specie.

However, studies on the neuropharmacological properties of the two major constituents of AcEO, 1N2PE and methyleugenol, have been identified. 1N2PE, isolated (93% pure) from *Dennettia tripetala* G. Baker EO, an African Annonaceae species, promoted hypnotic effect in doses between 100 and 400 mg/kg (ip) in mice, reducing sleep latency with potency higher than pentobarbitone sodium (50 mg/kg; ip) and diazepam (20 mg/kg; ip). It also promoted a dose-dependent increase in sleep time, reaching an effect equivalent to diazepam (standard drug) at 400 mg/kg. 1N2PE also promoted protection in the model of Pentylenetetrazol (PTZ)-induced convulsions in mice. At a dose of 20 mg/kg, 20% protection was observed, while at doses above 50 mg/kg, seizures were fully inhibited. Pretreatment with flumazenil (2 mg/kg; ip) blocked the protection provided by 1N2PE, indicating a likely involvement of γ-aminobutyric acid (GABA) pathways in its effect. Finally, evidence of an anxiolytic effect was also evaluated in the plus-maze model at doses between 5 and 20 mg/kg (ip) with equivalent potency to diazepam (20 mg/kg) (Oyemitan et al., 2013). Silva et al. (2014), in turn, demonstrated that 1N2PE, isolated from AcEO, is a strong acetylcholinesterase blocker, with potency equivalent to physostigmine. Its effect was related to the positioning of its nitro group next to the enzyme catalytic serine residue, forming a potent hydrogen bond with its hydroxyl group.

Methyleugenol, in turn, has been described as a central nervous system depressant, with anticonvulsant and anesthetic effects (Dallmeier Zelger et al., 1983; Sayyah et al., 2002). In this context Ding et al. (2014) demonstrates in cultured hippocampal neurons the agonist property of this substance on GABAa receptors. Considering the majority presence of these constituents in AcEO, they can be the basis of the traditional indications described as calming. However, there are still other properties to be investigated, such as the influence on neurodegeneration, memory, and depressive behaviors.

### Other Biological Activities

In addition to the effects addressed above, *A. canelilla* and its constituents have also been investigated for its antibiotic properties. Giongo et al. (2017), described the trypanomicidal effect of AcEO from bark, 1N2PE, and methyleugenol in a culture of *Trypanosoma evansi*, suggesting that may be a viable alternative for the treatment of this protozoosis. Silva et al. (2009), in turn, demonstrated the leishmanicidal effect of AcEO and 1N2PE on *Leishmania amazonensis*.

Antifungal activity has also been described. Evaluating EOs, hydrolates, ethanolic extract, and hexane and dichloromethane fractions of plant leaves or branches in the culture of bacteria (*Staphylococcus aureus* and *Pseudomonas aeruginosa*), human pathogenic fungi (*Candida albicans*), and phytopathogens (*Didymella bryoniae* and *Corynespora cassiicola*), reported that only EOs promoted growth inhibition and exclusively against *C. albicans* and *D. bryoniae*. These antifungal properties have been attributed mainly to 1N2PE (Oger et al., 1994; Silva, 2012).

## Potential for Aging-Related Diseases Treatment

**Figure 4:**
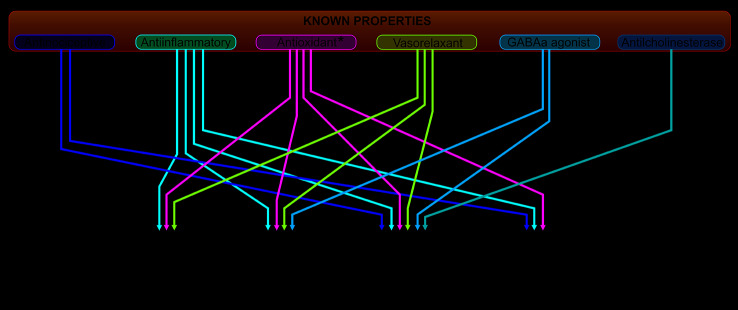
Prospects for assessing the therapeutic potential of *Aniba canelilla* in aging-related diseases, based on current knowledge. *Needs to be confirmed in biological models.

The human aging process involves the progressive alteration of the cell repair and proliferation capacity, in addition to nuclear and mitochondrial DNA alterations, constituting a progressively favorable context for the development of diseases. Among the events related to this senescence process is the so-called inflammating, a state caused by the increase in the production/release of inflammatory mediators and oxidative stress, caused by the imbalance between the production of ROS and the body's antioxidant capacity. The studies in this area discuss the cause and consequence relationships between the aging process and these two mechanisms, however, agree on the principle that they are key factors in the genesis and evolution of aging-related diseases, among which stands out the cancer, diabetes, and cardiovascular, respiratory, and neurological diseases (Khansari et al., 2009; Zuo et al., 2019) (**Figure 4**).

In this context, *A. canelilla* has great potential, as it brings together a set of advantageous activities for the treatment of clinical conditions related to inflammation and oxidative stress. The reviewed studies demonstrate the ability of the plant derivatives to modulate the inflammatory process by inhibiting pro-inflammatory and inducing anti-inflammatory cytokines, preventing edema formation and leukocyte migration to the focus of aggression (Lima, 2008; Choi et al., 2010; Vale et al., 2013). Based on these findings, much can be explored concerning the diversity of inflammatory diseases that can be benefited by their actions. Therefore, it is necessary to expand the studies on such actions, exploring its influences on the cellular inflammatory response, diapedesis mechanisms, and intracellular signaling pathways. Studies about its antioxidant properties, as previously mentioned, were limited to chemical DPPH tests (Silva et al., 2007; Silva, 2012; Mesquita et al., 2014; Martins et al., 2016), so we consider essential studies on its effects on ROS production, pro (NOX, NOS, XO, etc.) and antioxidant (Catalase, GPx, SOD, etc.) enzymes activity and macromolecules oxidation (lipid peroxidation, protein carbonylation, nucleic acid damage, etc.).

Regarding cardiovascular diseases (CVD), which emerge worldwide as the main cause of death (WHO, 2018), significant cardio modulatory and vasorelaxant effects of AcEO and its major constituent have been reported, leading to a reduction in blood pressure in hypertensive rats, through vago-vagal response and sGC stimulation, with consequent increase in cGMP production (Interaminense et al., 2010; Interaminense et al., 2013). It is important, however, to advance in the search for these effects. Antioxidant and anti-inflammatory properties of the plant could also promote additional benefits, reducing vascular remodeling, endothelial dysfunction, and atherosclerosis, which are induced/aggravated by inflammation and oxidative stress (Garcia et al., 2017; Zuo et al., 2019).

In view of the combination of vascular, anti-inflammatory, and antioxidant properties, we consider it is interesting to study its effects on the outcome of ischemic strokes (Kim et al., 2016). This condition is marked by rapid and intense neuronal death by apoptosis in ischemic core. In penumbra area, neuron death is triggered by glutamatergic excitotoxicity and oxidative stress, in addition to an important neuroinflammatory event, with the development of microgliosis and astrogliosis, and the release of pro-inflammatory cytokines (Fontes-Junior et al., 2016).

In fact, the interaction between oxidative stress and inflammation has been widely explored, identifying the interfaces between these mechanisms, that is, the state of “inflamming” increases the ROS production, favoring oxidative stress, in the same way as oxidative stress promotes the activation of inflammatory pathways and together are involved in chronic and degenerative conditions (Reuter et al., 2010). In addition, based on the evidence of the *A. canelilla* constituents action on central nervous system and traditional indications of use for the treatment of neurodegenerative conditions, such as Alzheimer's disease (AD), and depression (Rodrigues and Carlini, 2006; Madaleno, 2011), we consider it as a possible research line to be explored. The anticholinesterase effect attributed to 1N2PE may justify, at least in part, the use of *A. canelilla* for AD treatment, but there are no studies about its benefits on memory or disease progression, topic which assemble multiple mechanisms and must be elucidated in future studies. On behavior and emotionality, evidence of anxiolytic activity was observed (Oyemitan et al., 2013), but no study has yet explored traditional indications for depression treatment, considered an inflammatory disease too, or the supposed stimulating effect.

## Final Considerations

Studies on the aromatic species *A. canelilla*, popularly known as “Casca preciosa,” have ratified several of its traditional indications, showing its commercial and therapeutic potential, including antioxidant, antinociceptive, anti-inflammatory, anticholinesterase, anxiolytic, anticonvulsant, hypnotic, cardio modulatory, vasorelaxant, and antibiotic properties, through the application of its EO, extracts, and major constituents (1N2PE and Methyleugenol). However, it is necessary to advance in characterization of its mechanisms of action, pharmacokinetic, and clinical potential applications arising from each property. Special attention should also be paid to possible synergisms resulted from combined properties, such as antioxidant (needs to be confirmed in biological models), anti-inflammatory, and cardiovascular, which open new therapeutic possibilities on aging-related diseases, such as the treatment of cerebrovascular, neurobehavioral, and chronic degenerative diseases.

## Author Contributions

The review was conceived and designed by EF-J, FS-J, and DL-M. Data collection were performed by FS-J, DL-M, FP, MB, LF, and LQ. Data were analyzed by EF-J, FS-J, DL-M, CM, and JM. Drafting of the manuscript: FS-J, DL-M, FP, MB, LF, and LQ. Critical revision of the manuscript: EF-J, CM, and JM. All authors revised and approved the final version of the manuscript.

## Conflict of Interest

The authors declare that the research was conducted in the absence of any commercial or financial relationships that could be construed as a potential conflict of interest.

## Supplementary Material

Figure S1 Graphical abstract.
