# Supplementary figures and images for: Aniba canelilla (Kunth) Mez (Lauraceae): A Review of Ethnobotany, Phytochemical, Antioxidant, Anti-Inflammatory, Cardiovascular, and Neurological Properties
Source: Front Pharmacol. 2020 May 26;11:699. doi: 10.3389/fphar.2020.00699 (PMC7264103; doi:10.3389/fphar.2020.00699)

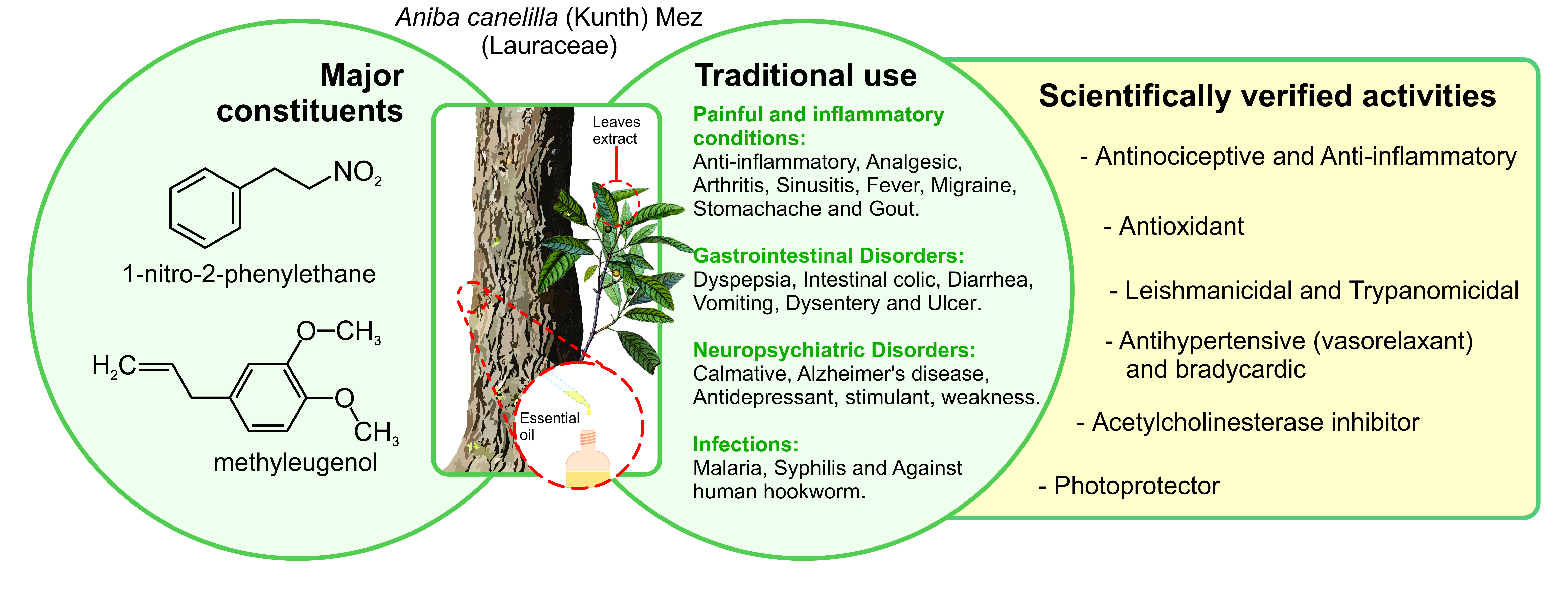

Supplement: Figure S1 — Graphical abstract. [file Image_1.jpeg]
